# Effect of Aripiprazole on Mismatch Negativity (MMN) in Schizophrenia

**DOI:** 10.1371/journal.pone.0052186

**Published:** 2013-01-07

**Authors:** Zhenhe Zhou, Hongmei Zhu, Lin Chen

**Affiliations:** Department of Psychiatry, Wuxi Mental Health Center of Nanjing Medical University, Wuxi, Jiangsu Province, China; Catholic University of Sacred Heart of Rome, Italy

## Abstract

**Background:**

Cognitive deficits are considered core symptoms of the schizophrenia. Cognitive function has been found to be a better predictor of functional outcome than symptom levels. Changed mismatch negativity (MMN) reflects abnormalities of early auditory processing in schizophrenia. Up to now, no studies for the effects of aripiprazole on MMN in schizophrenia have been reported.

**Methodology/Principal Findings:**

Subjects included 26 patients with schizophrenia, and 26 controls. Psychopathology was rated in patients with the Positive and Negative Syndrome Scale (PANSS) at baseline, after 4- and 8-week treatments with aripiprazole. Auditory stimuli for ERP consisted of 100 millisecond/1000 Hz standards, intermixed with 100 millisecond/1500 Hz frequency deviants and 250 millisecond/1000 Hz duration deviants. EEG was recorded at Fz. BESA 5.1.8 was used to perform data analysis. MMN waveforms were obtained by subtracting waveforms elicited by standards from waveforms elicited by frequency- or duration-deviant stimuli. Aripiprazole decreased all PANSS. Patients showed smaller mean amplitudes of frequency and duration MMN at baseline than did controls. A repeated measure ANOVA with sessions (i.e., baseline, 4- and 8-week treatments) and MMN type (frequency vs. duration) as within-subject factors revealed no significant MMN type or MMN type × session main effect for MMN amplitudes. Session main effect was significant. LSD tests demonstrated significant differences between MMN amplitudes at 8 weeks and those at both baseline and 4 weeks. There was significant negative correlation between changes in amplitudes of frequency and duration MMN and changes in PANSS total scores at baseline and follow-up periods.

**Conclusions:**

Aripiprazole improved the amplitudes of MMN. MMN offers objective evidence that treatment with the aripiprazole may ameliorate preattentive deficits in schizophrenia.

## Introduction

Cognitive deficits in many domains have been consistently replicated in patients with schizophrenia. These domains include the executive functions, perceptual and motor processing, attentional skills, vigilance, verbal learning and memory, spatial working memory, and verbal fluency [1]–[2]. These deficits are considered core symptoms of the schizophrenia. Most studies that link cognitive deficits to functional outcome in schizophrenia support the notion that neurocognitive function predicts social and occupational function. Measures of immediate memory, delayed memory, and executive function have been found to predict functional outcome with small to medium effect size [3]. Moreover, cognitive function has been found to be a better predictor of functional outcome than symptom levels [4]. Undoubtedly, the assessment of the level of cognitive functioning is regarded as crucial dimensions of the assessment of treatment outcomes in schizophrenia. Aripiprazole is a dopamine partial agonist approved for use in adults for short- and long-term treatment of schizophrenia and bipolar disorder. Aripiprazole shows a unique pharmacological profile. Via its partial agonism, it binds with high affinity to dopaminergic D2 and D3 receptors and serotonergic 5-HT1A receptors, and acts as an antagonist at 5-HT2A and 5-HT2C receptors [5]–[6]. The affinity of aripiprazole for muscarinergic and histaminergic H1 receptors is low. Strong dopamine D2 blockade and activity at the serotonin receptors as well as at postsynaptic D2 receptors might be responsible for its efficacy in reducing positive and somewhat negative symptoms, respectively; D2-receptor occupancy may be negatively correlated with certain types of learning, and aripiprazole' s partial-agonist activity may have implications for its effect on learning compared with typical antipsychotics [7].

Many studies used neurocognitive and functional measures, i.e., through the use of performance-based competence assessments as outcome measures in clinical trials to evaluate the effectiveness of aripiprazole in improving cognitive and functional outcomes. For example, in a previously randomized, open-label study that included 169 patients with schizophrenia or schizoaffective disorder, aripiprazole and olanzapine were associated with improvements from baseline in general cognitive functioning at week 8, with effects that remained relatively stable over 26 weeks; especially, aripiprazole-treated patients had a significant improvement in verbal learning from baseline to the 8- and 26-week assessments [8]. The authors suggested that the neurocognitive effects of aripiprazole were at least as good as those of olanzapine. Another study assessed the cognitive effects of aripiprazole in inpatients with schizophrenia [9]. Results showed aripiprazole significantly improveed PANSS total score and all subscores between baseline and endpoint visit, and patients improved significantly in verbal memory, reaction time and reaction quality/attention from baseline to week eight. Furthermore, mean z-values of individual cognitive domains summarized in a global cognitive index improved significantly from baseline to week eight.A study that used fMRI to assess the effect of aripiprazole and risperidone on cognitive function in n healthy volunteers indicated aripiprazole improved working memory compared to risperidone [10].

Cognitive deficits in schizophrenia not only include deficient performance in higher cognitive domains but also extend to information processing at the sensory and preattentive level [11]. The capacity of the human brain to detect deviance in the acoustic environment pre-attentively is reflected in a brain event-related potential (ERP), MMN. MMN is an effective measure of preattentive information processing by auditory change detection. MMN is a negative ERP component elicited by deviant stimulus, i.e. the standard sounds changed frequency, duration, intensity or location. Namely, the MMN is believed to be the outcome of a comparison process, commonly elicited when an incoming stimulus differs from the memory of repetitive tones (standards) occurring in the recent acoustic past. Because in the absence of such a memory trace no MMN is generated, MMN generation indexes auditory sensory memory and reflects context-dependent information processing at the level of the auditory sensory cortex. There is no agreement to date regarding whether MMN reflects an attention-independent process. A previous study originally proposed that the MMN was unaffected by various attentional manipulations [12]. The conclusion was based on evidence suggesting that it was the mismatch process between the neural trace of the standard (the repetitive sound) and the incoming deviant sound that generates the MMN response, which was unaffected. Several studies showed that MMN generation was affected by attention [13]–[15]. In addition, MMN likely reflects N-methyl-D-aspartate channel current influx in cortical layers II and III, based on animal and human experiments [16]. A study examined whether MMN-like activity occurs in epidural auditory cortex (AC) potentials in awake and anesthetized rats to high and low frequency and long and short duration deviant sounds [17]. ERPs to deviants were compared with ERPs to common standards and also with ERPs to deviants when interspersed with many different standards to control for background regularity effects. High frequency (HF) and long duration deviant ERPs in the awaked rat showed evidence of deviance detection, consisting of negative displacements of the deviant ERP relative to ERPs to both common standards and deviants with many standards. The HF deviant MMN-like response was also sensitive to the extent of regularity in recent acoustic stimulation. Anesthesia in contrast resulted in positive displacements of deviant ERPs. Results suggest that epidural MMN-like potentials to HF sounds in awake rats encode deviance in an analogous manner to the human MMN, laying the foundation for animal models of disorders characterized by disrupted MMN generation, such as schizophrenia.

To date, there have been many studies on MMN in schizophrenia. Several studies reported that MMN amplitude in response to tone duration and tone frequency deviants, respectively, was reduced in patients with schizophrenia [18]–[19]. One study showed that the presence of MMN abnormalities was related to illness onset [20]. However, another study displayed normal MMN generation in first-episode patients [21]. Thus, whether MMN deficits are present at illness onset remains controversial. Furthermore, MMN deficits are not observed in other major mental illnesses such as bipolar disorder or major depression suggesting that they may be fairly specific to schizophrenia [22]. A recent study showed MMN deficit of schizophrenia was replicated in a Han Chinese population, and the MMN was a better adjunctive diagnostic utility for schizophrenia [23]. In schizophrenia patients, glutathione dysregulation at the gene, protein and functional levels, leads to NMDA receptor hypofunction [16]. Because MMN is an auditory evoked potential component related to NMDA receptor function [24], deficits in NMDA receptor function can be quantitatively and non-invasively assessed through the measurement of MMN. A previous study had used N-acetyl-cysteine (NAC) to determine whether increased levels of brain glutathione would improve MMN in patients with schizophrenia [25]. The results displayed that treatment with NAC significantly improved MMN generation compared with placebo. It concluded that MMN enhancement may precede changes to indices of clinical severity, highlighting the possible utility ERPs as a biomarker of treatment efficacy.

Since changed MMN reflects abnormalities of early auditory processing in schizophrenia, we suppose that aripiprazole treatment may lead to the improvement of MMN. Up to now, no studies for the effects of aripiprazole on MMN in schizophrenia have been reported. The goal of the present study was to investigate whether the effects of aripiprazole on abnormalities of early auditory processing in patients with schizophrenia were reflected by auditory MMN.

## Materials and Methods

All research procedures were approved by the Ethics Committee of Nanjing Medical University, China on Human Studies and conducted in accordance with the Declaration of Helsinki. All participants gave written informed consent to participate. Because patients had a compromised capacity to consent, we gave all research procedures to their next of kins, care takers or guardians, and their next of kins, care takers or guardians consented on the behalf of participants whose capacity to consent was compromised.

### 2.1 Study Subjects

Subjects were 26 patients with a Diagnostic and Statistical Manual of Mental Disorders (4^th^ ed, DSM-IV) diagnosis criteria for schizophrenia, and 26 matched age and gender controls with no personal or family history of schizophrenia. Patients with schizophrenia were recruited from Wuxi Mental Heath Center of Nanjing Medical University in Jiangsu, China. Controls were recruited from the employees of Wuxi Mental Health Center of Nanjing Medical University. Subjects and controls were excluded from the study if they were smokers; or had a diagnosis of alcohol or substance dependence, neurological disorders, all kinds of head injury; or had received electroconvulsive therapy in the last six months. All participants were Chinese.

### 2.2 Clinical assessments

All participants underwent a clinical assessment by a psychiatrist to collect information on medication, socio-demographic data, and to confirm/exclude a DSM-IV diagnosis. On the day of the ERP recording, psychopathology was rated in patients with PANSS [26]. Handedness was assessed using the Annett handedness scale [27]. Ratings on this scale were recoded into the following definitions of handedness: Annett score (1) = right, (2–7) = mixed, (8) = left. Patients were treated with aripiprazole for 8 weeks. The effective rate, defined as a ≥30% reduction in score on PANSS overall scale from baseline, was calculated from the assessment after 8 week treatments. Safety and tolerability were assessed using the Treatment Emergent Symptom Scale (TESS) [28]. To ensure consistency and reliability of clinical assessments over time, training workshops were conducted in a regular manner to examine and re-examine inter-rater reliability coefficients. Coefficients of >0.80 averaged across the whole course of the study were achieved on PANSS overall scale and subscales.

The demographic characteristics of the sample are detailed in Table 1.

**Table 1:** Demographic characteristics of the sample.

	Patients	Controls
Sex ratio (M/F)	26 (14∶12)	26 (14∶12)
Mean age (S.D.)	33 (11)	33 (11)
Age range	18–59	18–59
Handedness		
*R/M/L*	15/8/3	14/9/3
*(% R/M/L)*	(58%/31%/11%)	(54%/35%/11%)

M: male. F: female. S.D.: standard deviation. R: right. M: mixed. L: left.

### 2.3 Experimental procedure

#### 2.3.1 Stimulation protocol and procedure

ERP recordings were acquired during the presentation of auditory stimuli. Auditory stimuli consisted of 100 milliseconds (ms)/1000 Hz standards intermixed with 100 ms/1500 Hz frequency deviants and 250 ms/1000 Hz duration deviants. All stimuli had a rise/fall time of 5 ms. Stimuli were presented in a fixed order (four standards, one frequency deviant, four standards, one duration deviant) with a stimulus onset asynchrony of 300 ms. The stimuli were presented through foam insert earphones at a nominal intensity of a 75 dB level. Stimuli were presented in four blocks with 1000 stimuli each totaling 4000 stimuli including 3200 standards, 400 frequency deviants and 400 duration deviants. During presentation of the auditory test paradigm, subjects watched a silent self-selected video film to divert attention from the tones, and to minimize boredom and reduce eye movement artifacts. Subjects were constantly monitored. Short breaks were offered to ensure full alertness and comfort during the recording session.

In order to detect the treatment effects on MMN, auditory ERPs were recorded at baseline, 4 weeks and 8 weeks of aripiprazole treatment. For healthy controls, ERPs were recorded once.

#### 2.3.2 Electroencephalographic recordings

According to the 10/20 International System, Electroencephalography (EEG) was recorded with the Stellate Harmonie EEG device (Physiotec Electronics Ltd. Canada) from Fz, left mastoid and right mastoid site using Electro-Cap Electrode System (ECI™ Electro-Caps, Electro-cap International, INL. U.S.A). Ear electrodes served as a reference and the ground electrode was attached to the forehead. Eye movement artifacts were monitored by recording vertical and horizontal electro oculogram (EOG) from electrodes placed above and below the right eye and at the left outer canthus. Electrode impedance was kept below 5 kΩ. System band pass was 0.1–30 Hz and digitalized continuously at a sampling rate of 250 Hz. Digital tags were obtained for all auditory stimuli.

### 2.4 Data analysis

Brain Electrical Source Analysis program (BESA, Version 5.1.8, Software) was used to perform data analysis. Epochs were constructed that consisted of a 100 ms pre-stimulus baseline and a 500 ms post-stimulus interval. All epochs with amplitudes exceeding ±75 µV at any electrode were excluded automatically. Epochs were averaged offline for each subject and stimulus type and digitally filtered with a low-pass filter of 15 Hz (24 dB down). MMN waveforms were obtained by subtracting waveforms elicited by standards, from waveforms elicited by frequency- or duration-deviant stimuli. Frequency MMN amplitude was defined as the peak negativity within a 100 to 300 ms latency window, and duration MMN amplitude was defined as the peak negativity within the 200 to 400 ms range.

### 2.5 Statistical Analyses

Data were analyzed using SPSS (version 10.0). Comparisons of PANSS scores (PANSS total scores, Comparisons of amplitudes of MMN between controls and patients were done using paired-sample t-tests. PANSS scores were analyzed by one-way repeated measure analysis of variances (ANOVA) with session (baseline, 4- and 8-week treatments) as within-subject factors. The effects of aripiprazole treatment on amplitudes of MMN were analyzed by repeated measure ANOVA with session (baseline, 4- and 8-week treatments) and MMN type (frequency vs. duration) as within-subject factors. Least square difference (LSD) tests were performed as post hoc analyses if indicated. Correlation coefficients between MMN and PANSS scores were calculated by the Pearson test. Alpha values of .05 were considered significant throughout.

## Results

### 3.1 Outcome of aripiprazole treatment

At baseline, 18 patients were neuroleptic naive, 8 neuroleptic free (3 for at least half a year, and 5 for at least 1 month). After 2 weeks of follow-up, patients received aripiprazole 20–30 mg/day (mean value 25.58, S.D. 3.83). The loss ratio of follow-up during the treatment is 0; the side effects included insomnia and anxiety (4 patients each, 15%), nausea (3 patients, 11.5%) and constipation (2 patient, 7.6%). 16 patients (61.5%) reported at least one side effect, while 10 patients (38.5%) had no side effects. Side effects were mild to moderate in severity. No combined medication for the therapy in 26 patients during the study.

### 3.2 Comparisons of PANSS before and after aripiprazole treatment

PANSS scores were analyzed by one-way repeated measure analysis of variances (ANOVA) with session (baseline, 4- and 8-week treatments) as within-subject factors revealed significant session main effect for PANSS scores (for PANSS total scores: F = 269, df = 2, p = 0.000; for Positive symptom scale scores: F = 185, df = 2, p = 0.000; for Negative symptom scale scores: F = 79, df = 2, p = 0.000; for Total psychopathology scale scores: F = 56, df = 2, p = 0.000;). Aripiprazole decreased all PANSS, total psychopathology, positive symptom and negative symptom scale scores. According to PANSS scores, the effective rate is 89%. There was significant negative correlation between changes in amplitudes of frequency and duration MMN and changes in PANSS total scores at baseline and follow-up periods(*r* = −0.39, −0.42, *P* = 0.012, 0.016 respectively).There was no significant correlation between changes in latencies of frequency and duration MMN and changes in PANSS total scores at baseline and follow-up periods. (Table 2).

**Table 2:** PANSS scores (presented as mean (SD)) before and after aripiprazole treatment.

	Baseline	After 4-week treatments	After 8-week treatments
PANSS (total scores)	96.3(12.5)	68.3(16.8)	62.7(18.4)
Positive symptom scale	22.4(10.2)	13.0(4.6)	11.0(4.7)
Negative symptom scale	26.3(13.0)	18.4(9.1)	16.9(11.7)
Total psychopathology scale	46.5(6.6)	37.0(11.2)	30.8(8.1)

### 3.3 Comparison at baseline of patients and controls

**Figure 1:**
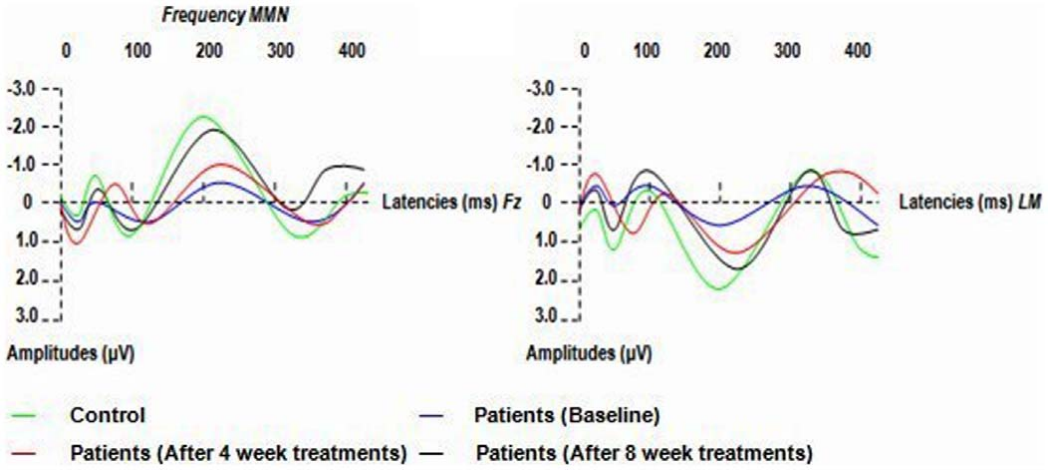
Grand averages of Frequency MMN (MMN waveforms were obtained by subtracting waveforms elicited by standards from waveforms elicited by frequency- deviant stimuli) in patients (at baseline, after 4- week and 8- week treatments of aripiprazole) and controls. LM: left mastoid.

**Figure 2:**
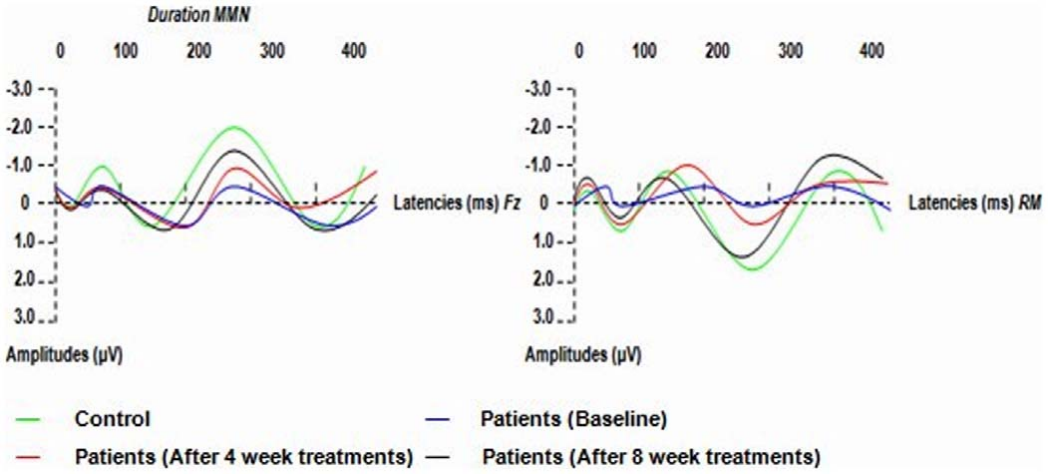
Grand averages of Duration MMN (MMN waveforms were obtained by subtracting waveforms elicited by standards from waveforms elicited by duration-deviant stimuli) in patients (at baseline, after 4- week and 8- week treatments of aripiprazole) and controls. RM: right mastoid.

Patients showed smaller mean amplitudes of frequency and duration MMN at baseline than did controls (in frequency MMN, t = 2.967, P = 0.001; in duration MMN, t = 3.363, P = 0.001; df for all electrodes = 25). The mean amplitudes of frequency and duration MMN were reduced in patients at 8- week treatments compared to controls (in frequency MMN, t = 1.832, P = 0.043; in duration MMN, t = 1.872, P = 0.039; df for all electrodes = 25). No differences in latencies between patients and controls were observed (Table 3; Fig. 1. and Fig. 2.).

**Table 3:** Amplitudes and latencies of MMN (presented as mean (SD)) of healthy controls (n = 26) and patients (n = 26) at baseline, 4- and 8- week treatments.

Types	Frequency		Duration	
Components	Latency, ms	Amplitude, µV	Latency, ms	Amplitude, µV
Controls	191.1(10.0)	2.13(0.5)	283.5(20.4)	2.18(0.9)
Patients (baseline)	195.7(11.6)	0.56(0.9)	289.2(21.5)	0.59(0.8)
Patients (after 4- week treatments)	193.3(15.4)	0.91(0.6)	285.9(22.7)	0.90(0.9)
Patients (after 8- week treatments)	190.0(17.8)	1.89(0.8)	284.2(19.6)	1.92(0.5)

### 3.4 Effects of aripiprazole treatment

A repeated measure ANOVA with session (baseline, 4- and 8-week treatments) and MMN type (frequency vs. duration) as within-subject factors revealed no significant MMN type or MMN type ×session main effect for MMN amplitudes (for MMN type: F = 0.852, df = 1, p = 0.476; for MMN type ×session: F = 0.419, df = 2, p = 0.746). Session main effect, however, was significant (F = 4.470, df = 2, p = 0.028). LSD tests were performed as post hoc analyses and demonstrated significant differences between MMN amplitudes at 8 weeks and those at both baseline (p = 0.001) and 4 weeks (p = 0.039). MMN amplitudes at 8 weeks were higher than those at 4 weeks and those at baseline; There was a significant difference between MMN amplitudes at 4 weeks and those at baseline (p = 0.045). MMN amplitudes at 4 weeks were higher than those at baseline (Table 3; Fig. 1. and Fig. 2.).

## Discussion

This study is the first to employ electrophysiological indices of automatic auditory information processing, i.e., MMN, to assess cognitive improvement in patients with schizophrenia treated by atypical neuroleptic aripiprazole. Our study replicates the findings of numerous studies that demonstrated the presence of neurocognitive deficits in patients with schizophrenia on most domains tested, including attention, vigilance, and immediate memory, working memory, delayed memory and executive function [1].

Our trial results authenticate previous hypotheses that treatment with aripiprazole leads to the improvement of MMN in schizophrenia. Aripiprazole is a new antipsychotic with a unique receptor binding profile that combines partial agonistic activity at D2 receptor and 5-HT 1A receptor and potent antagonism at 5-HT 2A receptor. This receptor profile makes it possible for it to act as a dopamine system stabilizer. Because MMN amplitude is highest in frontal channels [29]–[31], MMN amplitudes at Fz electrodes present the status of cognitive function. Our study showed that aripiprazole improved the amplitudes of MMN after 4-week treatments, especially, with the prolonging treatment period the improvement of MMN is significant. Above results prove that aripiprazole has an effect on passive attention in patients with schizophrenia. From a neuroelectrophysical standpoint, MMN offers objective evidence that treatment with the aripiprazole ameliorates preattentive deficits in schizophrenia.

A previous study showed reduced MMN in stable chronic patients with schizophrenia, and deduced that chronic patients represent a more homogenous sample concerning the genetics of MMN deficits [11]. Another study displayed that MMN amplitude was reduced in patients with schizophrenia and relatives compared with controls, and there were no significant differences between patients and relatives, therefore, the results suggest that reduced MMN amplitude may be an endophenotype marker of the predisposition to schizophrenia [32]. Above two studies support that MMN deficits may represent a trait marker. However, a study reported that the MMN has no a significant familial influence and is normal among the unaffected relatives. The researchers concluded that although the MMN is abnormal in patients with schizophrenia, it is a weak or unreliable marker of vulnerability when applied to subclinical populations. Therefore it is unlikely to be an endophenotype for the disorder [33].

Representing a later stage of auditory processing, the mismatch negativity (MMN) reflects the detection of deviations from an auditory regularity. Inconsistent with our study, a recent research reported that normal frequency MMN and reduced duration MMN amplitudes and significantly shorter duration MMN latencies were observed in patients with schizophrenia [34]. The discrepancies might be attributed to the fact that the described ERPs of two studies are differently modified by factors such as stimulus parameters, attention, arousal, or psychotropic medication.

In our study, the mean amplitudes of frequency and duration MMN were reduced in patients after 4- week treatments compared to controls, which deduces that MMN abnormalities are state-dependent. However, because of the small sample our results have to be considered preliminary. As a matter of fact, whether MMN deficits represent a trait marker or state marker remains controversial. The probable reason for inconsistencies is that different stimulus condition designs were used and insufficient numbers of subjects were recruited for these studies.

Neurocognitive impairment is now recognized as a fundamental symptom of schizophrenia [35]–[36]. Improvement in cognitive function is increasingly recognized as an important goal of therapy. To some extent, the degree of neurocognitive impairment has been shown to be a much stronger predictor of community functioning than either positive or negative symptom severity [37]. A study investigated the longitudinal stability of mismatch negativity deficits and their longitudinal relationship to poor functional status [38]. Results indicated that MMN deficits and their relationship to poor functional status are stable over time in patients with chronic schizophrenia, suggesting that MMN may be useful for assessing medication response and other factors in longitudinal studies.

In this trial, aripiprazole had significant effects on amplitudes of MMN after 4-week treatments. The results differed from that of other atypical antipsychotics, such as clozapine, risperidone and olanzapine [39]–[41]. Consistent with the previous study, the cognitive profile of aripiprazole differs from that of other atypical antipsychotics in schizophrenia patients [42].

In the past, MMN for both duration and frequency deviants was investigated in patients with schizophrenia, and the results showed that patients with schizophrenia demonstrated significantly smaller mean MMN than did healthy control subjects [22]. We observed significant correlation between changes in amplitudes of frequency and duration MMN and changes in PANSS scores at baseline and follow-up periods. Consistent with previous research, the present study showed that MMN amplitudes were significantly reduced in the patient group, which demonstrated that MMN might be an abnormal index of preattentive automatic auditory information processing. Furthermore, MMN amplitude improvement may be a possible biomarker of treatment efficacy. The improvement of this functional marker may indicate an important pathway towards new therapeutic strategies that target cognitive dysfunction in schizophrenia. It is important that clinicians understand the benefits and limitations of modern neuroimaging techniques and are also suitably equipped to appraise future developments [43].

In conclusion, the use of MMN in evaluating psychopathology and therapeutic effects is helpful in the clinical management of schizophrenic patients. Therefore, it is necessary to validate this study effect using similar parameters in future studies.

## Supporting Information

Strobe Checklist S1 **STROBE Statement.**
(DOC)
