# Supplementary material for: Effect of Aripiprazole on Mismatch Negativity (MMN) in Schizophrenia
Source: PLoS One. 2013 Jan 7;8(1):e52186. doi: 10.1371/journal.pone.0052186 (PMC3538635; doi:10.1371/journal.pone.0052186)
Supplement: Strobe Checklist S1 — STROBE Statement. (DOC) [file pone.0052186.s001.doc]

**Supporting Information**

**Strobe Check List S1**

STROBE Statement—**Effect of Aripiprazole on Mismatch Negativity (MMN) in Schizophrenia**

|  | Item No |  |
| --- | --- | --- |
| **Title and abstract** | 1 | **Title: Effect of Aripiprazole on Cognitive Function in Schizophrenia ：A Mismatch Negativity Potentials Study** |
|  |  | **Abstract**  **Background:** Cognitive deficits are considered core symptoms of the schizophrenia. Cognitive function has been found to be a better predictor of functional outcome than symptom levels. Changed MMN reflects abnormalities of early auditory processing in schizophrenia. Up to now, no studies for the effects of aripiprazole on MMN in schizophrenia have been reported.  **Methodology/Principal Findings:** Subjects included 26 patients with schizophrenia, and 26 controls. Psychopathology was rated in patients with the PANSS at baseline, after 4- and 8-week treatments with aripiprazole. Auditory stimuli for ERP consisted of 100 millisecond/1000 Hz standards, intermixed with 100 millisecond/1500 Hz frequency deviants and 250 millisecond/ 1000 Hz duration deviants. EEG was recorded at Fz. BESA 5.1.8 was used to perform data analysis. MMN waveforms were obtained by subtracting waveforms elicited by standards from waveforms elicited by frequency- or duration-deviant stimuli. Aripiprazole decreased all PANSS**.** Patients showed smaller mean amplitudes of frequency and duration MMN at baseline than did controls. A repeated measure ANOVA with sessions (i.e., baseline, 4- and 8-week treatments) and MMN type (frequency vs. duration) as within-subject factors revealed no significant MMN type or MMN type ×session main effect for MMN amplitudes. Session main effect was significant. LSD tests demonstrated significant differences between MMN amplitudes at 8 weeks and those at both baseline and 4 weeks. There was significant negative correlation between changes in amplitudes of frequency and duration MMN and changes in PANSS total scores at baseline and follow-up periods.  **Conclusions:** Aripiprazole improved the amplitudes of MMN. MMN offers objective evidence that treatment with the aripiprazole may ameliorate preattentive deficits in schizophrenia**.** |
| Introduction | | |
| Background/rationale | 2 | **Background:** Cognitive deficits are considered core symptoms of the schizophrenia. Cognitive function has been found to be a better predictor of functional outcome than symptom levels. Changed MMN reflects abnormalities of early auditory processing in schizophrenia. Up to now, no studies for the effects of aripiprazole on MMN in schizophrenia have been reported. |
| Objectives | 3 | Since changed MMN reflects abnormalities of early auditory processing in schizophrenia, we suppose that aripiprazole treatment may lead to the improvement of MMN. Up to now, no studies for the effects of aripiprazole on MMN in schizophrenia have been reported. The goal of the present study was to investigate whether the effects of aripiprazole on abnormalities of early auditory processing in patients with schizophrenia were reflected by auditory MMN. |
| Methods | | |
| Study design | 4 | A case-controlled observational experiment. |
| Setting | 5 | The experiment was completed in the Department of psychology at Wuxi Mental Health Center, China, from July 2011 to March 2012. |
| Participants | 6 | Subjects were 26 patients with a Diagnostic and Statistical Manual of Mental Disorders (4^th^ ed, DSM-IV) diagnosis criteria for schizophrenia, and 26 matched age and gender controls with no personal or family history of schizophrenia. Patients with schizophrenia were recruited from Wuxi Mental Heath Center of Nanjing Medical University in Jiangsu, China. Controls were recruited from the employees of Wuxi Mental Health Center of Nanjing Medical University. Subjects and controls were excluded from the study if they were smokers; or had a diagnosis of alcohol or substance dependence, neurological disorders, all kinds of head injury; or had received electroconvulsive therapy in the last six months. All participants were Chinese. |
| Variables | 7 | On the day of the ERP recording, psychopathology was rated in patients with the Positive and Negative Syndrome Scale (PANSS). Handedness was assessed using the Annett handedness scale. Ratings on this scale were recoded into the following definitions of handedness: Annett score (1) = right, (2–7) = mixed, (8) = left.  In order to detect the treatment effects on MMN, auditory ERPs were recorded at baseline, 4 weeks and 8 weeks of aripiprazole treatment. For healthy controls, ERPs were recorded once. At baseline, 18 patients were neuroleptic naive, 8 neuroleptic free (3 for at least half a year, and 5 for at least 1 month). After 2 weeks of follow-up, patients received aripiprazole 20-30 mg/day (mean value 25.58, S.D. 3.83)  MMN waveforms were obtained by subtracting waveforms elicited by standards, from waveforms elicited by frequency- or duration-deviant stimuli. Frequency MMN amplitude was defined as the peak negativity within a 100 to 300 ms latency window, and duration MMN amplitude was defined as the peak negativity within the 200 to 400 ms range. |
| Data sources/ measurement | 8* | **Clinical assessments**  All participants underwent a clinical assessment by a psychiatrist to collect information on medication, socio-demographic data, and to confirm/exclude a DSM-IV diagnosis. On the day of the ERP recording, psychopathology was rated in patients with PANSS. Handedness was assessed using the Annett handedness scale. Ratings on this scale were recoded into the following definitions of handedness: Annett score (1) = right, (2–7) = mixed, (8) = left. Patients were treated with aripiprazole for 8 weeks. The effective rate, defined as a ≥ 30% reduction in score on PANSS overall scale from baseline, was calculated from the assessment after 8 week treatments. Safety and tolerability were assessed using the Treatment Emergent Symptom Scale (TESS). To ensure consistency and reliability of clinical assessments over time, training workshops were conducted in a regular manner to examine and re-examine inter-rater reliability coefficients. Coefficients of > 0.80 averaged across the whole course of the study were achieved on PANSS overall scale and subscales.  **Stimulation protocol and procedure**  ERP recordings were acquired during the presentation of auditory stimuli. Auditory stimuli consisted of 100 milliseconds (ms)/1000 Hz standards intermixed with 100 ms/1500 Hz frequency deviants and 250 ms/1000 Hz duration deviants. All stimuli had a rise/fall time of 5 ms. Stimuli were presented in a fixed order (four standards, one frequency deviant, four standards, one duration deviant) with a stimulus onset asynchrony of 300 ms. The stimuli were presented through foam insert earphones at a nominal intensity of a 75 dB level. Stimuli were presented in four blocks with 1000 stimuli each totaling 4000 stimuli including 3200 standards, 400 frequency deviants and 400 duration deviants. During presentation of the auditory test paradigm, subjects watched a silent self-selected video film to divert attention from the tones, and to minimize boredom and reduce eye movement artifacts. Subjects were constantly monitored. Short breaks were offered to ensure full alertness and comfort during the recording session.  In order to detect the treatment effects on MMN, auditory ERPs were recorded at baseline, 4 weeks and 8 weeks of aripiprazole treatment. For healthy controls, ERPs were recorded once. At baseline, 18 patients were neuroleptic naive, 8 neuroleptic free (3 for at least half a year, and 5 for at least 1 month). After 2 weeks of follow-up, patients received aripiprazole 20-30 mg/day (mean value 25.58, S.D. 3.83)  **lectroencephalographic recordings**  According to the 10/20 International System, Electroencephalography (EEG) was recorded with the Stellate Harmonie EEG device (Physiotec Electronics Ltd. Canada) from Fz, left mastoid and right mastoid site using Electro-Cap Electrode System (ECI^TM^ Electro-Caps, Electro-cap International, INL. U.S.A). Ear electrodes served as a reference and the ground electrode was attached to the forehead. Eye movement artifacts were monitored by recording vertical and horizontal electro oculogram (EOG) from electrodes placed above and below the right eye and at the left outer canthus. Electrode impedance was kept below 5 kΩ. System band pass was 0.1 - 30 Hz and digitalized continuously at a sampling rate of 250 Hz. Digital tags were obtained for all auditory stimuli.  **Data analysis**  Brain Electrical Source Analysis program (BESA, Version 5.1.8, Software) was used to perform data analysis. Epochs were constructed that consisted of a 100 ms pre-stimulus baseline and a 500 ms post-stimulus interval. All epochs with amplitudes exceeding ±75 μV at any electrode were excluded automatically. Epochs were averaged offline for each subject and stimulus type and digitally filtered with a low-pass filter of 15 Hz (24 dB down). MMN waveforms were obtained by subtracting waveforms elicited by standards, from waveforms elicited by frequency- or duration-deviant stimuli. Frequency MMN amplitude was defined as the peak negativity within a 100 to 300 ms latency window, and duration MMN amplitude was defined as the peak negativity within the 200 to 400 ms range.  **Statistical Analyses**  Data were analyzed using SPSS (version 10.0). Comparisons of PANSS scores (PANSS total scores, Comparisons of amplitudes of MMN between controls and patients were done using paired-sample t-tests. PANSS scores were analyzed by one-way repeated measure analysis of variances (ANOVA) with session (baseline, 4- and 8-week treatments) as within-subject factors. The effects of aripiprazole treatment on amplitudes of MMN were analyzed by repeated measure ANOVA with session (baseline, 4- and 8-week treatments) and MMN type (frequency vs. duration) as within-subject factors. Least square difference (LSD) tests were performed as post hoc analyses if indicated. Correlation coefficients between MMN and PANSS scores were calculated by the Pearson test. Alpha values of .05 were considered significant throughout. |
| Bias | 9 | No |
| Study size | 10 | According to experimental design, subjects included 26 patients with schizophrenia, and 26 controls. |
| Quantitative variables | 11 | Comparisons of PANSS scores (PANSS total scores, Comparisons of amplitudes of MMN between controls and patients were done using paired-sample t-tests. PANSS scores were analyzed by one-way repeated measure analysis of variances (ANOVA) with session (baseline, 4- and 8-week treatments) as within-subject factors. The effects of aripiprazole treatment on amplitudes of MMN were analyzed by repeated measure ANOVA with session (baseline, 4- and 8-week treatments) and MMN type (frequency vs. duration) as within-subject factors. Least square difference (LSD) tests were performed as post hoc analyses if indicated. Correlation coefficients between MMN and PANSS scores were calculated by the Pearson test. Alpha values of .05 were considered significant throughout. |
| Statistical methods | 12 | (*a*) Data were analyzed using SPSS (version 10.0). Comparisons of PANSS scores (PANSS total scores, Comparisons of amplitudes of MMN between controls and patients were done using paired-sample t-tests. PANSS scores were analyzed by one-way repeated measure analysis of variances (ANOVA) with session (baseline, 4- and 8-week treatments) as within-subject factors. The effects of aripiprazole treatment on amplitudes of MMN were analyzed by repeated measure ANOVA with session (baseline, 4- and 8-week treatments) and MMN type (frequency vs. duration) as within-subject factors. Least square difference (LSD) tests were performed as post hoc analyses if indicated. Correlation coefficients between MMN and PANSS scores were calculated by the Pearson test. Alpha values of .05 were considered significant throughout. |
|  |  | (*b*) PANSS scores were analyzed by one-way repeated measure analysis of variances (ANOVA) with session (baseline, 4- and 8-week treatments) as within-subject factors. The effects of aripiprazole treatment on amplitudes of MMN were analyzed by repeated measure ANOVA with session (baseline, 4- and 8-week treatments) and MMN type (frequency vs. duration) as within-subject factors. Least square difference (LSD) tests were performed as post hoc analyses if indicated. Correlation coefficients between MMN and PANSS scores were calculated by the Pearson test. A |
|  |  | (*c*) no missing data were addressed |
|  |  | (*d*) Subjects were 26 patients with a Diagnostic and Statistical Manual of Mental Disorders (4^th^ ed, DSM-IV) diagnosis criteria for schizophrenia, and 26 matched age and gender controls with no personal or family history of schizophrenia. |
| Results | | |
| Participants | 13* | (a)  ***Patients Controls***  Sex ratio (M/F) 26 (14:12) 26 (14:12)  Mean age (S.D.) 33 (11) 33 (11)  Age range 18– 59 18– 59  Handedness  R/M/L 15 / 8 / 3 14 / 9 / 3  (% R/M/L) (58%/31%/11%) (54%/35%/11%)  (M: male. F: female. S.D.: standard deviation. R: right. M: mixed. L: left.) |
|  |  | (b) All 26 subjects and controls were anticipated this study |
| Descriptive data | 14* | (a)  ***Patients Controls***  Sex ratio (M/F) 26 (14:12) 26 (14:12)  Mean age (S.D.) 33 (11) 33 (11)  Age range 18– 59 18– 59  Handedness  R/M/L 15 / 8 / 3 14 / 9 / 3  (% R/M/L) (58%/31%/11%) (54%/35%/11%)  (M: male. F: female. S.D.: standard deviation. R: right. M: mixed. L: left.) |
|  |  | (b) no missing data were addressed |
| Outcome data | 15* | All 26 subjects and controls were anticipated this study |
| Main results | 16 | **Outcome of aripiprazole treatment**  At baseline, 18 patients were neuroleptic naive, 8 neuroleptic free (3 for at least half a year, and 5 for at least 1 month). After 2 weeks of follow-up, patients received aripiprazole 20-30 mg/day (mean value 25.58, S.D. 3.83). The loss ratio of follow-up during the treatment is 0; the side effects included insomnia and anxiety (4 patients each, 15%), nausea (3 patients, 11.5%) and constipation (2 patient, 7.6%). 16 patients (61.5%) reported at least one side effect, while 10 patients (38.5%) had no side effects. Side effects were mild to moderate in severity. No combined medication for the therapy in 26 patients during the study.  **Comparisons of PANSS before and after aripiprazole treatment**  PANSS scores were analyzed by one-way repeated measure analysis of variances (ANOVA) with session (baseline, 4- and 8-week treatments) as within-subject factors revealed significant session main effect for PANSS scores (for PANSS total scores: F=269, df=2, p=0.000; for Positive symptom scale scores: F=185, df=2, p=0.000; for Negative symptom scale scores: F=79, df=2, p=0.000; for Total psychopathology scale scores: F=56, df=2, p=0.000;). Aripiprazole decreased all PANSS, total psychopathology, positive symptom and negative symptom scale scores. According to PANSS scores, the effective rate is 89%. There was significant negative correlation between changes in amplitudes of frequency and duration MMN and changes in PANSS total scores at baseline and follow-up periods(*r* = -0.39, -0.42, *P* = 0.012, 0.016 respectively).There was no significant correlation between changes in latencies of frequency and duration MMN and changes in PANSS total scores at baseline and follow-up periods.  **Comparison at baseline of patients and controls**  Patients showed smaller mean amplitudes of frequency and duration MMN at baseline than did controls (in frequency MMN, t=2.967, P=0.001; in duration MMN, t=3.363, P=0.001; df for all electrodes=25). The mean amplitudes of frequency and duration MMN were reduced in patients at 8- week treatments compared to controls (in frequency MMN, t=1.832, P=0.043; in duration MMN, t=1.872, P=0.039; df for all electrodes=25). No differences in latencies between patients and controls were observed.  **Effects of aripiprazole treatment**  A repeated measure ANOVA with session (baseline, 4- and 8-week treatments) and MMN type (frequency vs. duration) as within-subject factors revealed no significant MMN type or MMN type ×session main effect for MMN amplitudes (for MMN type: F=0.852, df=1, p=0.476; for MMN type ×session: F=0.419, df=2, p=0.746). Session main effect, however, was significant (F=4.470, df=2, p=0.028). LSD tests were performed as post hoc analyses and demonstrated significant differences between MMN amplitudes at 8 weeks and those at both baseline (p=0.001) and 4 weeks (p=0.039). MMN amplitudes at 8 weeks were higher than those at 4 weeks and those at baseline; There was a significant difference between MMN amplitudes at 4 weeks and those at baseline (p=0.045). MMN amplitudes at 4 weeks were higher than those at baseline. |

| Other analyses | 17 | no |
| --- | --- | --- |
| Discussion | | |
| Key results | 18 | This study is the first to employ electrophysiological indices of automatic auditory information processing, i.e., MMN, to assess cognitive improvement in patients with schizophrenia treated by atypical neuroleptic aripiprazole. Our study replicates the findings of numerous studies that demonstrated the presence of neurocognitive deficits in patients with schizophrenia on most domains tested, including attention, vigilance, immediate memory, working memory, delayed memory and executive function.  Our trial results authenticate previous hypotheses that treatment with aripiprazole leads to the improvement of MMN in schizophrenia.  We observed significant correlation between changes in amplitudes of frequency and duration MMN and changes in PANSS scores at baseline and follow-up periods. Consistent with previous research, the present study showed that MMN amplitudes were significantly reduced in the patient group, which demonstrated that MMN might be an abnormal index of preattentive automatic auditory information processing. Furthermore, MMN amplitude improvement may be a possible biomarker of treatment efficacy. The improvement of this functional marker may indicate an important pathway towards new therapeutic strategies that target cognitive dysfunction in schizophrenia. It is important that clinicians understand the benefits and limitations of modern neuroimaging techniques and are also suitably equipped to appraise future developments [38].  In conclusion, the use of MMN in evaluating psychopathology and therapeutic effects is helpful in the clinical management of schizophrenic patients. Therefore, it is necessary to validate this study effect using similar parameters in future studies. |
| Limitations | 19 | Limitation of the study is the small sample. Because of the small sample, our results have to be considered preliminary. As a matter of fact, whether MMN deficits represent a trait marker or state marker remains controversial. The probable reason for inconsistencies is that different stimulus condition designs were used and insufficient numbers of subjects were recruited for these studies. |
| Interpretation | 20 | Our trial results authenticate previous hypotheses that treatment with aripiprazole leads to the improvement of MMN in schizophrenia. Aripiprazole is a new antipsychotic with a unique receptor binding profile that combines partial agonistic activity at D2 receptor and 5-HT 1A receptor and potent antagonism at 5-HT 2A receptor. This receptor profile makes it possible for it to act as a dopamine system stabilizer. Because MMN amplitude is highest in frontal channels, MMN amplitudes at Fz electrodes present the status of cognitive function. Our study showed that aripiprazole improved the amplitudes of MMN after 4-week treatments, especially, with the prolonging treatment period the improvement of MMN is significant. Above results prove that aripiprazole has an effect on passive attention in patients with schizophrenia. From a neuroelectrophysical standpoint, MMN offers objective evidence that treatment with the aripiprazole ameliorates preattentive deficits in schizophrenia.  A previous study showed reduced MMN in stable chronic patients with schizophrenia, and deduced that chronic patients represent a more homogenous sample concerning the genetics of MMN deficits. Another study displayed that MMN amplitude was reduced in patients with schizophrenia and relatives compared with controls, and there were no significant differences between patients and relatives, therefore, the results suggest that reduced MMN amplitude may be an endophenotype marker of the predisposition to schizophrenia. Above two studies support that MMN deficits may represent a trait marker. However, a study reported that the MMN has no a significant familial influence and is normal among the unaffected relatives. The researchers concluded that although the MMN is abnormal in patients with schizophrenia, it is a weak or unreliable marker of vulnerability when applied to subclinical populations. Therefore it is unlikely to be an endophenotype for the disorder. In our study, the mean amplitudes of frequency and duration MMN were reduced in patients after 4- week treatments compared to controls, which deduces that MMN abnormalities are state-dependent. However, because of the small sample our results have to be considered preliminary. As a matter of fact, whether MMN deficits represent a trait marker or state marker remains controversial. The probable reason for inconsistencies is that different stimulus condition designs were used and insufficient numbers of subjects were recruited for these studies.  Neurocognitive impairment is now recognized as a fundamental symptom of schizophrenia. Improvement in cognitive function is increasingly recognized as an important goal of therapy. To some extent, the degree of neurocognitive impairment has been shown to be a much stronger predictor of community functioning than either positive or negative symptom severity. In this trial, aripiprazole had significant effects on amplitudes of MMN after 4-week treatments. The results differed from that of other atypical antipsychotics, such as clozapine, risperidone and olanzapine. Consistent with the previous study, the cognitive profile of aripiprazole differs from that of other atypical antipsychotics in schizophrenia patients.  In the past, MMN for both duration and frequency deviants was investigated in patients with schizophrenia, and the results showed that patients with schizophrenia demonstrated significantly smaller mean MMN than did healthy control subjects. We observed significant correlation between changes in amplitudes of frequency and duration MMN and changes in PANSS scores at baseline and follow-up periods. Consistent with previous research, the present study showed that MMN amplitudes were significantly reduced in the patient group, which demonstrated that MMN might be an abnormal index of preattentive automatic auditory information processing. Furthermore, MMN amplitude improvement may be a possible biomarker of treatment efficacy. The improvement of this functional marker may indicate an important pathway towards new therapeutic strategies that target cognitive dysfunction in schizophrenia. It is important that clinicians understand the benefits and limitations of modern neuroimaging techniques and are also suitably equipped to appraise future developments. |
| Generalisability | 21 | Consistent with previous research, the present study showed that MMN amplitudes were significantly reduced in the patient group, which demonstrated that MMN might be an abnormal index of preattentive automatic auditory information processing. Furthermore, MMN amplitude improvement may be a possible biomarker of treatment efficacy. The improvement of this functional marker may indicate an important pathway towards new therapeutic strategies that target cognitive dysfunction in schizophrenia. It is important that clinicians understand the benefits and limitations of modern neuroimaging techniques and are also suitably equipped to appraise future developments.  In conclusion, the use of MMN in evaluating psychopathology and therapeutic effects is helpful in the clinical management of schizophrenic patients. Therefore, it is necessary to validate this study effect using similar parameters in future studies. |
| Other information | | |
| Funding | 22 | This study was supported by: the Medical Research Foundation of Department of Public Health, Jiangsu Province, China. No.H201043 |
